# Response to 'Factors associated with depressive symptoms among adolescents in Indonesia: A cross-sectional study of results from the Indonesia Family Life Survey'

**DOI:** 10.51866/lte.26-00057

**Published:** 2026-10-13

**Authors:** Haerawati Idris, Fatimah Tuzzahra

**Affiliations:** 1 Faculty of Public Health, Sriwijaya University, Raya Palembang KM 32., Indralaya, Ogan Ilir, South Sumatra, Indonesia.

**Keywords:** Adolescent, Depression, Chronic Disease, Cross-Sectional Studies, Indonesia

**Dear Editor,**

We sincerely thank the author for their thoughtful and constructive comments regarding our article entitled ‘Factors associated with depressive symptoms among adolescents in Indonesia: A crosssectional study of results from the Indonesia Family Life Survey’.^1,2^ We appreciate their recognition of the importance of our findings and their suggestions regarding the interpretation of the association between chronic illness and depressive symptoms. We respond to the three points raised as follows:

First, regarding the broad classification of chronic illness, we agree that chronic illness represents a heterogeneous group of conditions that may differ substantially in severity, duration, functional limitations, treatment burden and psychosocial consequences. In our study, chronic illness history was defined according to the information available in the Indonesia Family Life Survey (IFLS) and included several conditions, including asthma, tuberculosis, cardiovascular disease, kidney disease, gastrointestinal disorders and cancer. Therefore, our analysis intended to examine the overall association between a history of chronic illness and depressive symptoms rather than disease- specific associations.

The systematic review by Hards et al. found that evidence on the relationship between depression and self-evaluation among adolescents with chronic illness remains limited and suggested that this relationship may differ across chronic conditions.^3^ Our study revealed that a history ofchronic illness showed the strongest significant association with depressive symptoms among the variables included in the final multivariable model (adjusted odds ratio=2.781; 95% confidence interval=2.308–3.350). However, we emphasise that this finding should be interpreted as presenting the strongest observed association among the variables included in our model, rather than evidence that chronic illness is the most important causal determinant of depressive symptoms. Future studies using datasets containing disease-specific information, including the type, duration, severity, treatment burden and functional impairment of chronic illness, would allow a more clinically meaningful assessment of these associations.

Second, regarding family and psychosocial determinants, we agree that factors such as parental mental health, family psychiatric history, adverse childhood experiences, family functioning, peer relationships and social support may be relevant to adolescent mental health and could potentially influence the association between chronic illness and depressive symptoms. However, these variables were not available in the raw IFLS data used for our analysis in a form that could be incorporated into the present multivariable model. Consequently, our selection of covariates was necessarily restricted to variables available in the dataset and measured sufficiently for inclusion in the analysis. We therefore acknowledge that the absence of these potentially relevant family and psychosocial variables represents an important limitation and that residual confounding cannot be excluded.

Importantly, we do not interpret the observed association between chronic illness and depressive symptoms as evidence that chronic illness acts independently of family or psychosocial factors. Rather, we showed that the adolescents with a history of chronic illness had higher odds of developing depressive symptoms after adjustments for the measured covariates available in the IFLS dataset. Thus, the association should be interpreted cautiously and should not be regarded as a causal effect.

We have therefore clarified this limitation and agree that future research should incorporate more comprehensive family and psychosocial measures. Such research would be particularly valuable in identifying whether these factors confound, mediate or modify the relationship between chronic illness and depressive symptoms. Given the complexity of these relationships, longitudinal studies would be better suited to examining these pathways over time.

Third, regarding the interpretation of causality and the possibility of a bidirectional relationship, we fully agree that the cross-sectional design of our study does not permit the determination of temporal sequence or causal direction. Our analysis identified factors associated with depressive symptoms at the time ofthe survey but could not establish whether chronic illness preceded depressive symptoms, whether depressive symptoms influenced subsequent health-related behaviours and physical health or whether both were influenced by other underlying factors.

The possibility of a bidirectional relationship is clinically and scientifically plausible. The longitudinal evidence synthesised by Zheng et al. indicates that depressive symptoms among adolescents with chronic illness may subsequently be associated with poorer disease control, reduced self-management and medication adherence, lower quality of life and increased hospitalisation.^4^ However, the review also emphasises that longitudinal evidence remains limited across populations with different chronic illnesses. Therefore, we agree that prospective studies are needed to establish temporal relationships and clarify the mechanisms linking chronic illness, depressive symptoms and health-related behaviours.

We would also like to clarify that our description of chronic illness as the ‘strongest factor’ referred specifically to the magnitude of the significant association observed in our multivariable model. It was not intended to imply causal priority or suggest that chronic illness is necessarily the primary determinant of adolescent depression. We agree that this distinction is important when interpreting findings from a cross-sectional observational study.

Finally, we appreciate the author’s comments regarding the clinical and public health implications of our findings. We agree that adolescents living with chronic medical conditions may represent an important population for attention to mental health. Evidence from longitudinal research supports consideration of mental health care alongside chronic disease management, particularly because depressive symptoms may adversely affect disease-related outcomes and quality of life. Nevertheless, our cross-sectional findings alone do not establish the effectiveness of specific screening strategies or integrated care models. Such interventions should be evaluated in appropriately designed implementation and longitudinal studies.

## Conclusion

We appreciate the author’s constructive comments. We agree that future studies should distinguish individual chronic conditions, incorporate family and psychosocial characteristics that were unavailable in our dataset and employ longitudinal designs to clarify temporal and potentially bidirectional relationships. These approaches would provide a more comprehensive understanding of depressive symptoms among Indonesian adolescents and help inform the development of appropriate prevention and intervention strategies.
